# Diagnosing tuberculosis in hospitalized HIV-infected individuals who cannot produce sputum: is urine lipoarabinomannan testing the answer?

**DOI:** 10.1186/s12879-017-2914-7

**Published:** 2017-12-28

**Authors:** Natasha F. Sabur, Aliasgar Esmail, Mantaj S. Brar, Keertan Dheda

**Affiliations:** 1 Lung Infection and Immunity Unit, Division of Pulmonology and University of Cape Town Lung Institute, Department of Medicine, University of Cape Town, H47 Old Main Building, Groote Schuur Hospital, Observatory, Cape Town, 7925 South Africa; 2 Division of Respirology, Department of Medicine, St. Michael’s Hospital and West Park Healthcare Centre, University of Toronto, Toronto, Canada; 3 Department of Surgery, Mount Sinai Hospital, University of Toronto, Toronto, Canada; 4 Institute of Infectious Diseases and Molecular Medicine, University of Cape Town, Cape Town, South Africa

**Keywords:** Urine LAM, Tuberculosis, Sputum-scarce, HIV

## Abstract

**Background:**

Up to one third of HIV-infected individuals with suspected TB are sputum-scarce. The Alere Determine™ TB LAM Ag lateral flow strip test can be used to diagnose TB in HIV-infected patients with advanced immunosuppression. However, how urine LAM testing should be incorporated into testing algorithms and in the context of specific patient sub-groups remains unclear.

**Methods:**

This study represents a post hoc sub-group analysis of data from a randomized multi-center parent study. The study population consisted of hospitalized HIV-infected patients with suspected TB who were unable to produce sputum and who underwent urine LAM testing. The diagnostic utility of urine LAM for TB in this group was compared to the performance of urine LAM in patients who did produce a sputum sample in the parent study.

**Results:**

There were a total of 187 and 2341 patients in the sputum-scarce and sputum-producing cohorts, respectively. 80 of the sputum-scarce patients underwent testing with urine LAM. In comparison to those who did produce sputum, sputum-scarce patients had a younger age, a lower Karnofsky performance score, and a lower weight and BMI at admission. A greater proportion of sputum-scarce patients were urine LAM positive, compared to those who were able to produce sputum (31% vs. 21%, *p* = 0.04). A higher proportion of sputum-scarce patients died within 8 weeks of admission (32% vs. 24%, *p* = 0.013). We inferred that 19% of HIV-infected sputum-scarce patients suspected of TB were diagnosed with tuberculosis by urine LAM testing, with an estimated positive predictive value of 63% (95% CI 43–82%).

**Conclusions:**

Urine LAM testing can effectively identify tuberculosis in HIV-infected patients who are at a higher risk of mortality yet are unable to generate a sputum sample for diagnostic testing. Our findings support the use of urine LAM testing in sputum-scarce hospitalized HIV-infected patients, and its incorporation into diagnostic algorithms for this patient population.

## Background

Despite many recent advances in the diagnosis of tuberculosis (TB), it is estimated that one third of all TB cases are missed (either not diagnosed or not reported) [1, 2]. Current diagnostic tests for pulmonary TB rely on generation of a sputum sample. Historically, sputum microscopy, where sputum is examined under microscopy for acid-fast bacilli, has been the first-line test for TB. The World Health Organization (WHO) now advocates for the Xpert MTB/RIF test, an automated real-time polymerase chain reaction assay for detection of *M. tuberculosis* and rifampicin resistance, [3] as the initial diagnostic test for all adults and children with suspected TB [4]. The Xpert MTB/RIF test has demonstrated improved accuracy over smear microscopy, and has resulted in an improvement in time to treatment initiation for TB patients [5, 6]. However, despite allowing for more rapid diagnostic results as well as information on first line drug resistance, the Xpert MTB/RIF assay also requires the patient to generate a sputum sample of adequate quality and volume for this test to yield a result.

The HIV epidemic has changed the face of tuberculosis, particularly in Africa, where nearly 40% of active TB cases are co-infected with HIV [2]. In this population, TB often does not produce cavities and sputum has a low bacillary load [7], rendering a significant proportion of patients smear-negative [8–10], and up to a third of patients unable to produce sputum for diagnostic testing [11]. Various methods of sputum acquisition including sputum induction and bronchoscopy have been tested in this population, but these methods are expensive and require special facilities [12, 13]. Additionally, they require significant processing time and often do not provide same-day diagnosis, which has particular relevance in high-burden settings where many patients fail to return for results and are therefore often lost to follow-up [14]. Moreover, in hospitalized HIV-infected patients, post-mortem studies have illustrated a large burden of undiagnosed TB [15], and in this population rapid initiation of TB treatment may reduce mortality [1, 16, 17].

Lipoarabinomannan (LAM), an immunogenic glycolipid component of the bacterial cell wall excreted in the urine, offers a unique method for detection of *M. tuberculosis* [18–20] and has been recently shown to reduce mortality in hospitalized HIV patients when used to guide TB treatment initiation [21]. Urine LAM testing would likely have particular utility in HIV patients who are unable to produce a sputum sample, as urine specimens are generally easily obtained. Although the WHO has published guidelines about the use of urine LAM, they do not address how the test should be used in diagnostic algorithms when alternative tests are available (ex. Xpert MTB/RIF, smear microscopy, culture, etc.), or which patient sub-groups would benefit from specific first-line testing strategies [22]. To our knowledge, the performance of urine LAM specifically in sputum-scarce HIV patients has not previously been evaluated. To address this knowledge gap, we evaluated the diagnostic utility of urine LAM testing in a cohort of hospitalized HIV-infected patients unable to generate an adequate sputum sample for conventional TB testing.

## Methods

### Study design

This study represents a post hoc sub-group analysis of data from a pragmatic, randomized, parallel arm, multi-center study with stratified randomization by country. The primary analysis has already been reported [21]. The study was approved by the appropriate national regulatory authorities and by the University of Cape Town Human Research Ethics Committee. All patients provided informed written consent in their first language. This trial was registered with Clinicaltrials.gov (https://clinicaltrials.gov/ct2/show/NCT01770730).

### Patient enrollment and randomization

Patients admitted to ten urban or peri-urban hospitals in South Africa, Tanzania, Zambia and Zimbabwe were screened for study inclusion. A detailed description of hospital care, HIV and TB prevalence, routine clinical care, and the diagnostics infrastructure at each hospital are previously reported. [21] The inclusion criteria included: i) HIV-infected persons; ii) at least one of the following symptoms: current fever or cough, drenching night sweats, or self-reported loss-of-weight; iii) illness severe enough to necessitate hospitalization; iv) age ≥ 18 years; v) granting of informed consent. The exclusion criteria included: i) patients receiving any anti-TB medication in the 60 days prior to testing, and ii) unable to provide at least 30mls urine. Eligible patients were randomized to receive standard available TB diagnostics at each center or standard TB diagnostics plus adjunctive LAM, using centralized computer-generated allocation lists, stratified by country. The patients and the study team were not masked to both allocation and test results. In addition to a urine specimen, patients were asked to expectorate a minimum of two sputa for routine TB diagnosis. For patients unable to self-expectorate, sputum induction was employed. Clinical assessment by the attending physicians and chest x-ray (CXR) facilities were available in most cases, however additional radiology and non-sputum sampling was differentially available in study hospitals and requesting these investigations was at the discretion of the attending clinical team. For patients in the study arm, Alere Determine™ TB LAM Ag lateral flow strip test testing was performed at the bedside, according to manufacturer’s instructions. A grade 2 cutoff point or higher was deemed as a positive urine LAM result. The attending clinical team made all decisions regarding patient therapy and initiation of anti-TB treatment and the timing thereof, including acting upon the LAM test results. The WHO guidelines for the treatment of smear-negative tuberculosis were routinely used at study hospitals.

### Outcomes and statistical analysis

The goal of this analysis was to assess the diagnostic utility of urine LAM for TB in HIV-infected patients admitted for suspected TB and who were unable to produce an adequate sputum sample. As no reference standard exists for the diagnosis of TB in the absence of a sputum sample, the findings of the LAM test were compared to the performance in patients who did produce a sputum sample in this study. Exact binomial 95% confidence intervals were calculated for proportions, and differences in proportions were calculated with 95% confidence intervals (using a normal-approximation) for comparisons between the groups. For categorical variables, differences between groups were evaluated using chi-square tests. For continuous variables, differences in means were calculated and t-tests were employed to evaluate differences in means between groups (applying the central-limit theorem to non-normal distributions).

## Results

### Patients

Of the 2528 patients enrolled in the study, a total of 222 patients did not have a sputum result. 35 of these patients generated a sputum sample but no result was recorded; therefore, these patients were not included in the sputum-scarce cohort. The remaining 187 patients (7.4% of the randomized patients) were unable to produce a sputum sample despite the availability of sputum induction facilities at all hospital sites.

### Baseline characteristics

In this sputum-scarce cohort of 187 patients, the mean age was 36 years and 51% were female. In comparison to those who did produce a sputum sample, there was evidence that these patients were younger, had a lower Karnofsky performance score, and had a lower weight and BMI at admission (Table 1). In addition, there was evidence of an association with being unable to produce sputum and recruitment from Zambia, as a disproportionate number of sputum-scarce patients were recruited from this site. There was no evidence of a difference in baseline CD4+ count (Table 1).

**Table 1:** Baseline characteristics in HIV-infected persons stratified by ability to produce a sputum sample

	Sputum-Scarce *N* = 187	Sputum-Producing *N* = 2341	*p*-value
Mean age in years (SD)	36.3 (9.6)	37.9 (10.5)	0.031
Sex distribution M:F (% males)	92:95 (49%)	1136:1205 (49%)	0.86
Mean CD4+ Count in cells/mm^3^ (SD)	178 (241)	150 (176)	0.15
Mean Karnofsky Score (SD)	50.6 (12.4)	53.4 (16.1)	0.005
Mean weight in Kg (SD)	47.8 (9.1)	53.4 (12.0)	<0.001
Mean BMI (SD)	17. 2 (3.3)	19.5 (4.4)	<0.001

### Outcomes

Among the sputum-scarce cohort of 187 patients, 80 patients were assigned to the LAM study arm of the parent study and had urine LAM testing performed. A greater proportion of patients in this group were urine LAM positive, compared to those who were able to produce a sputum sample (31% vs. 21%, *p* = 0.04, Table 2). In addition, a greater proportion of patients underwent CXR (88% vs. 55%, *p* < 0.001) and were felt by the treating clinician to have a CXR suggestive of tuberculosis, compared to those who produced sputum (59% vs. 48%, *p* = 0.010). However, there was no evidence that the proportion of patients who received treatment for TB varied by the ability to produce a sputum sample (48% vs. 49%, *p* = 0.68). A higher proportion of those unable to produce sputum died within 8 weeks of admission (32% vs. 24%, *p* = 0.013).

**Table 2:** Diagnostic findings, treatment, and outcomes, stratified by ability to produce a sputum sample

	Sputum-scarce *N* = 187	Sputum-producing *N* = 2341	*p*-value
Positive urine LAM (%)	25/80^a^ (31%)	250/1173 (21%)	0.038
Sputum culture positive		1691/2306 (27%)	
Chest X-ray performed	164/187 (88%)	1286/2341 (55%)	<0.001
CXR suggestive of TB	96/164 (59%)	615/1286 (48%)	0.010
Received TB treatment	89/186 (48%)	1157/2340 (49%)	0.68
Death within 8 weeks	60/187 (32%)	560/2341 (24%)	0.013

^a^A total of 80 patients in the sputum-scarce cohort were randomized to the LAM arm in the parent study and had a urine LAM test performed

Due to the lack of a reference standard for the diagnosis of TB in patients who cannot produce sputum, we used the diagnostic accuracy of urine LAM testing in those able to produce sputum and applied the false-positive rate to this cohort (using sputum culture positivity as the reference standard for the diagnosis of TB). This assumes the specificity of the urine LAM test does not differ among the patient populations that are able and unable to produce sputum, as CD4+ counts did not differ between these groups. Among those who were able to produce sputum, were sputum culture negative, and underwent LAM testing, 11.8% were found to have a positive urine LAM result (100/848, 95% CI 9.6–14.1%), which we considered the false positive rate of the test. Among those unable to produce sputum and who underwent a urine LAM test, 31% were found to have a positive urine LAM test (25/80, 95% CI 21%–43%). The difference between these two proportions was used to estimate the true positive rate of urine LAM testing in those unable to produce a sputum sample, and was estimated at 19% (95% CI 9.0–30%); i.e. 19% of HIV-infected patients suspected of TB but unable to produce a sputum specimen are diagnosed with TB with the use of a urine LAM test. Under these assumptions, the positive predictive value of a positive urine LAM test in this cohort is estimated as 63% (95% CI 43–82%).

## Discussion

In this study, we identified a group of hospitalized, HIV-infected, sputum-scarce patients in whom diagnosis of tuberculosis was particularly challenging, and in whom a missed diagnosis could have had fatal consequences. Compared to the larger cohort of patients with suspected TB who were able to provide a sputum sample for diagnostic testing, this group had a lower weight and Karnofsky performance status, illustrating significant chronic illness and functional impairment. Sputum-scarce patients had a higher proportion of urine LAM test positivity as well as a significantly higher mortality at 8 weeks post-hospitalization. We estimate that urine LAM correctly identified TB disease in nearly 20% of these patients, in whom a diagnosis of tuberculosis would otherwise have been potentially impossible without more invasive testing, and many of whom may not have been empirically treated for TB as inferred from published post-mortem studies [15, 23–25]. Interestingly, in our study, there was no concordance between CXR and urine LAM results, highlighting the difficulty in using CXR alone as a diagnostic modality in HIV-infected individuals given the wide array of chest pathology often seen in this patient population.

Our results illustrate the capability of urine LAM as a point-of-care test to diagnose TB in sputum-scarce patients. While it is now understood that a very high burden of undiagnosed tuberculosis exists in hospitalized HIV-infected patients [15], very little is known about those who are unable to produce a sputum sample, and as they are generally excluded from clinical studies, data on this group is limited. Certainly, smear negative TB patients are known to have significant diagnostic delays as well as greater morbidity and mortality compared to smear positive patients, in whom a diagnosis can more easily be achieved [26–28] and in this population, urine LAM used in conjunction with sputum smear microscopy has demonstrated benefit in diagnosing culture-confirmed TB [29]. The diagnostic accuracy of urine LAM is greatest in HIV-infected patients with advanced immunosuppression, likely representing disseminated TB and renal involvement of tuberculosis. [30] This study highlights the value of urine LAM in this population who, due to an inability in producing a sputum specimen, would otherwise remain undiagnosed and as a consequence, likely have a higher mortality.

In resource-limited settings, many such patients are started on empiric TB treatment, a strategy that is supported by the World Health Organization for resource-constrained areas with a high HIV prevalence. [31] However, this may unnecessarily expose patients to a long course of potentially toxic treatment, and although this strategy has recently demonstrated a potential survival benefit [32], it preceded the widespread availability of other diagnostic tools such as Xpert MTB/RIF testing. Even worse, patients often do not receive empiric treatment, evidenced by post-mortem studies demonstrating that up to 50% of patients with autopsy evidence of tuberculosis are not on treatment for TB at the time of death. [23] Our results suggest that urine LAM testing could add an important tool in the diagnostic armamentarium permitting diagnosis and appropriate treatment in hospitalized HIV-infected patients who are sputum-scarce.

Although sputum could not be obtained in only 7.4% of the patients randomized in the parent study, this is likely an underestimate of the problem of sputum scarcity in HIV-infected patients, since sputum induction facilities were available at all recruitment sites as part of the study protocol. A higher proportion of HIV-infected patients presenting to hospital with symptoms of tuberculosis are likely to be sputum-scarce [12], and in most resource-limited settings where induction facilities are unavailable, urine LAM may have broader applications. In addition, 12.3% of patients screened for inclusion into the parent study were too sick to provide informed consent, and therefore were excluded from study participation. This group of patients were likely to have also been too sick to provide sputum samples for diagnostic testing, and thereby represent an additional population in whom urine LAM may be most beneficial in achieving a diagnosis of TB.

To our knowledge, this is the first study to demonstrate the utility of urine LAM testing in patients with suspected TB who are unable to provide a sputum sample for diagnostic testing. The implications of this finding are important – sputum-scarce patients present a significant challenge to clinicians and TB control programs alike, and highlight deficiencies in current diagnostic algorithms that rely on a patient’s ability to generate a sputum sample. Urine LAM testing in this group may facilitate rapid diagnosis of tuberculosis and enable prompt treatment initiation in this very vulnerable group of patients. It also has implications for designing diagnostic algorithms when both Xpert MTB/RIF and urine LAM are available, as is the case in many TB and HIV endemic countries, and suggests that urine LAM should be the first port of call in sputum-scarce patients. Thus, our findings inform clinical practice and patient management strategies.

This study has some important limitations. The cohort of patients unable to generate a sputum sample was small, and a majority of the sputum-scarce patients were disproportionately recruited in selected countries, which could reflect local problems with sputum collection and induction facilities. However, patients in the sputum-scarce cohort had different clinical characteristics compared to the larger group of patients who were able to provide a sputum sample, suggesting that this group truly represents a unique subset of patients. In order to calculate the positive predictive value of urine LAM in the sputum-scarce group, we had to make the assumption that the specificity of the urine LAM test does not differ between patients who are able and unable to produce sputum.

## Conclusions

This study estimates that urine LAM testing has a high positive predictive value in hospitalized HIV-infected patients who are unable to generate sputum, and that urine LAM may identify patients with TB who would otherwise be missed and who have a high mortality. This point-of-care test acts as a powerful tool to aid the diagnosis of TB in a particularly challenging and vulnerable patient group, in whom a missed diagnosis has fatal consequences. This study supports the use of urine LAM testing in sputum-scarce, hospitalized HIV-infected patients, and should appropriately be incorporated into diagnostic algorithms.

## Abbreviations

BMI: body mass index
CXR: chest x-ray
HIV: human immunodeficiency virus
LAM: lipoarabinomannan
TB: tuberculosis
WHO: World Health Organization
